# A Telemonitoring and Hybrid Virtual Coaching Solution “CAir” for Patients with Chronic Obstructive Pulmonary Disease: Protocol for a Randomized Controlled Trial

**DOI:** 10.2196/20412

**Published:** 2020-10-22

**Authors:** Christoph Gross, Dario Kohlbrenner, Christian F Clarenbach, Adam Ivankay, Thomas Brunschwiler, Yves Nordmann, Florian v Wangenheim

**Affiliations:** 1 Department of Management, Technology, and Economics ETH Zürich Zürich Switzerland; 2 Department of Pulmonology University Hospital of Zürich Zürich Switzerland; 3 Ecole Polytechnique Fédérale de Lausanne Lausanne Switzerland; 4 IBM Research, Zürich Rüschlikon Switzerland; 5 docdok.health Ltd. Basel Switzerland

**Keywords:** protocol, randomized controlled trial, chronic obstructive pulmonary disease, telemonitoring, virtual coaching, disease management, chatbot, conversational agents

## Abstract

**Background:**

Chronic obstructive pulmonary disease (COPD) is one of the most common disorders in the world. COPD is characterized by airflow obstruction, which is not fully reversible. Patients usually experience breathing-related symptoms with periods of acute worsening and a substantial decrease in the health-related quality-of-life. Active and comprehensive disease management can slow down the progressive course of the disease and improve patients’ disabilities. Technological progress and digitalization of medicine have the potential to make elaborate interventions easily accessible and applicable to a broad spectrum of patients with COPD without increasing the costs of the intervention.

**Objective:**

This study aims to develop a comprehensive telemonitoring and hybrid virtual coaching solution and to investigate its effects on the health-related quality of life of patients with COPD.

**Methods:**

A monocentric, assessor-blind, two-arm (intervention/control) randomized controlled trial will be performed. Participants randomized to the control group will receive usual care and a CAir Desk (custom-built home disease-monitoring device to telemonitor disease-relevant parameters) for 12 weeks, without feedback or scores of the telemonitoring efforts and virtual coaching. Participants randomized to the intervention group will receive a CAir Desk and a hybrid digital coaching intervention for 12 weeks. As a primary outcome, we will measure the delta in the health-related quality of life, which we will assess with the St. George Respiratory Questionnaire, from baseline to week 12 (the end of the intervention).

**Results:**

The development of the CAir Desk and virtual coach has been completed. Recruitment to the trial started in September 2020. We expect to start data collection by December 2020 and expect it to last for approximately 18 months, as we follow a multiwave approach. We expect to complete data collection by mid-2022 and plan the dissemination of the results subsequently.

**Conclusions:**

To our knowledge, this is the first study investigating a combination of telemonitoring and hybrid virtual coaching in patients with COPD. We will investigate the effectiveness, efficacy, and usability of the proposed intervention and provide evidence to further develop app-based and chatbot-based disease monitoring and interventions in COPD.

**Trial Registration:**

ClinicalTrials.gov identifier: NCT04373070; https://clinicaltrials.gov/ct2/show/NCT04373070

**International Registered Report Identifier (IRRID):**

DERR1-10.2196/20412

## Introduction

### Background

Chronic obstructive pulmonary disease (COPD) is a chronic lung disease commonly caused by exposure to noxious particles (primarily from smoking) and characterized by respiratory symptoms (ie, dyspnea, cough, and sputum) and airflow obstruction [1]. Patients experience persistent respiratory symptoms with periods of acute worsening (exacerbations) and a substantial decrease in health-related quality of life (HRQOL). In 2016, about 251 million people worldwide were affected by COPD [2]*.* COPD is projected to become the third-leading global cause of death by 2030 [3]. In addition to the health implications, COPD causes a substantial economic burden on the individual and the health care systems [4]. There is no cure for COPD. However, effective treatment such as medication, smoking cessation, and physical exercise helps to relieve symptoms and prevent exacerbations [5]. Active disease management to reduce the burden of COPD and improve HRQOL therefore plays a crucial role in the therapeutic strategy.

Effective COPD management plans follow a patient-centered approach and aim to (1) prevent disease progression through the reduction of risk factors, (2) improve exercise tolerance, (3) improve health status, and (4) relieve symptoms [5]. Best practice treatment of COPD should be multidimensional and include medical care, self-management strategies, physical activity coaching, and behavioral change approaches [6-8]. One of these evidence-based disease management programs is *Living well with COPD* [9,10]. The program was developed by a Canadian consortium and is based on a disease self-management approach that covers educational topics as well as lifestyle coaching and physical activity advice supervised by health care professionals [9]. A randomized clinical trial showed that compared to usual care in a multicenter setup, the *Living well with COPD* intervention program reduced hospital admissions due to COPD exacerbations, emergency department visits, and unscheduled physician visits and improved HRQOL significantly [9]. A recent clinical trial conducted in Switzerland confirmed these results [8]. The implementation of the program, however, is both time and cost intensive.

Technological progress and the digitalization of medicine have the potential to make comprehensive interventions such as the *Living well with COPD* program easily accessible and applicable to a broad spectrum of patients with COPD without increasing the costs of the intervention. Prior research has proven the effectivity and efficiency of novel treatment solutions that incorporate telemonitoring and virtual coaching [11]. To our knowledge, however, no clinical trial has been conducted with a multimodal intervention based on the concept of *Living well with COPD* that is delivered remotely.

To fill this gap in research, we, a consortium between IBM Research, docdok.health, the University Hospital Zurich, and the Swiss Federal Institute of Technology developed a comprehensive telemonitoring and hybrid virtual coaching solution called CAir, which is based on the *Living well with COPD* program, and will investigate its effects on the HRQOL in patients with COPD.

### Hypotheses

The primary outcome of this study is the assessment of patients’ change in HRQOL, which we will evaluate with the St. George Respiratory Questionnaire (SGRQ) [12], a survey specifically designed for patients with chronic airflow limitations. A minimal clinically important difference of an average SGRQ score decrease of 4 units is associated with a slightly effective intervention, 8 units with a moderately effective intervention, and 12 units with a very effective intervention in patients with COPD [13].

Against this backdrop and considering the comprehensiveness of CAir, the hypotheses of this study are as follows:

1. More than 80% of the intervention group will have a ≥4-point change in SGRQ.
2. More than 40% of the intervention group will have a ≥8-point change in SGRQ.
3. More than 20% of the intervention group will have a ≥12-point change in SGRQ.
4. Approximately 10% of the control group will have a ≥4-point change in SGRQ.

We formulated the fourth hypothesis based on the Hawthorne effect [14], which states that individuals change their behaviour when they know they are being observed. This assumption is further supported by several studies in which the mean HRQOL within the control group also increased in a clinically relevant manner without any intervention [15].

## Methods

We will perform a monocentric, assessor-blind, two-arm (intervention/control) randomized controlled trial. The allocation ratio between the intervention and control group will be 2:1. Bias will be minimized through randomization, allocation concealment, assessor blinding, and statistical adjustment or subgrouping during the analysis. The study is expected to have an overall duration of 18 months (first patient in, last patient out). The length of study per patient will be 12 weeks. The recruitment period will last several months.

### Participants

#### Eligibility Criteria

Participants will be recruited from the Swiss population affected by COPD. Inclusion and exclusion criteria are outlined in Textbox 1.

**Inclusion and exclusion criteria and re-screening for the CAir study.**

**Inclusion criteria:**

- Provision of written informed consent
- Age≥40 years
- Ability to speak German fluently
- Diagnosis of chronic obstructive pulmonary disease according to the Global Initiative for Chronic Obstructive Lung Disease guidelines

**Exclusion criteria:**

- Physical or intellectual impairment precluding informed consent or protocol adherence
- Acute or recent (within the last 6 weeks) exacerbation of chronic obstructive pulmonary disease
- Attending a pulmonary rehabilitation program within the last 3 months
- Ongoing pregnancy

**Re-screening**

- In case of an exacerbation less than 6 weeks prior to the study or completing a pulmonary rehabilitation less than 3 months previous to the study, patients can be included with a delay to ensure eligibility

#### Recruitment, Screening, and Informed Consent Procedure

Participants will be recruited directly from the outpatient clinic of the Department of Pulmonology of the University Hospital Zurich by a physician or study coordinator involved in the care, and indirectly by screening the hospital’s patient database for the eligibility and ineligibility criteria (Textbox 1). In case patients meet the criteria, they will be contacted by a letter of enquiry that contains a study information sheet and a prestamped reply envelope. Those indicating interest in taking part in the study will be contacted by phone. In addition, cooperating pulmonary clinics and primary care physicians will equally screen their databases for patients fulfilling the main inclusion criteria (ie, diagnosis of COPD and age ≥40 years) and will send them study participation requests. Interested patients will then independently contact the study team of the University Hospital Zurich, which will evaluate the remaining inclusion and exclusion criteria.

We will not offer any material compensation for participating in this study. Potential participants will be informed about the scientific benefit of their participation; however, no other incentives will be offered.

All patients will be required to provide written informed consent before starting the trial. In that context, investigators will educate each participant about the nature of the study, its purpose, procedures involved, expected duration, potential risks and benefits, and any possible discomfort. Patients will also be informed that their participation is strictly voluntary and that they can withdraw from the study at any time without stating any reason. They will also be informed that withdrawing from the study will not affect subsequent medical assistance and treatment. The participants will be further advised that their medical records may be examined by authorized individuals other than their treating physician. Participants will be given sufficient time to reflect extensively on their participation.

The consent form will be signed and dated by the investigator or designee immediately following the signature of the participant. The consent form will be retained as part of the study records. A signed copy will be provided to each study participant.

#### Sample Size

Sample size calculations used a power of 80% and a two-sided significance level of 0.05. To increase usability reporting and account for possible dropouts, we decided to use a 2:1 (intervention:randomization) ratio and doubled the result of the sample size calculation. The sample size calculation yielded a total of 42 patients (28 intervention, 14 control). This sample size will result in an effect size of 0.9 to detect a clinically relevant between-group difference in the SGRQ.

### Technological Study Setup

**Figure 1:**
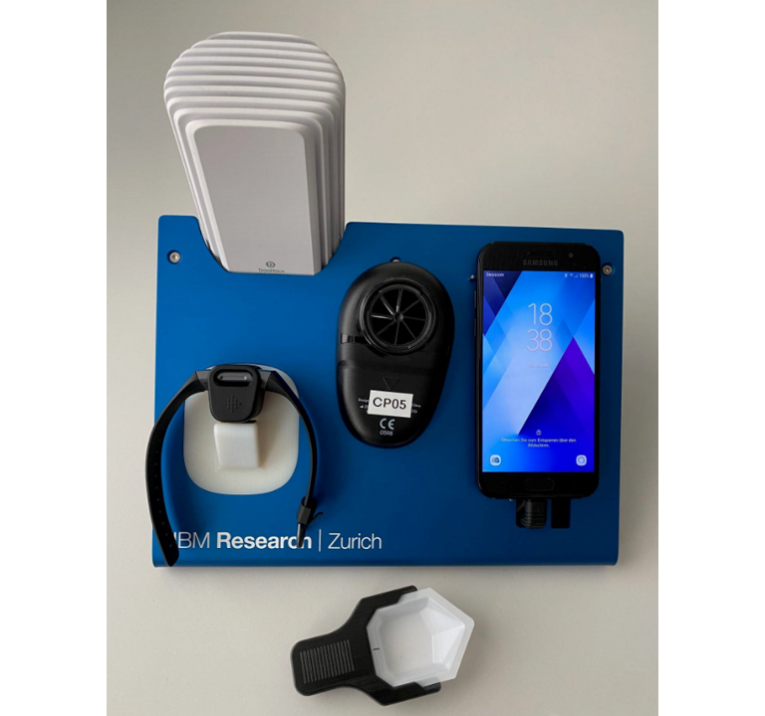
CAir Desk with sensors.

Considering proper charging and data transfer, the daily usage of multiple connected devices can be very demanding for some individuals. Thus, a selection of sensors was combined into the CAir Desk to improve usability and compliance. The CAir Desk can be placed next to the patient's bed and allows charging of all devices with a single power plug (Figure 1).

The smartphone serves as not only the user interface to enter questionnaires and access the symptom history through the docdok app, but also a data transfer hub from the devices to the CAir database in the IBM Cloud. Patient symptoms and the environment are tracked through a home spirometer (Air Next Spirometer, NuvoAir) to capture lung function (forced expiratory volume in 1 second/forced vital capacity), a wearable physical activty tracker (Charge 3, Fitbit Inc) to report vital signs as well as the number of daily steps as a proxy for physical activity, and the microphone and camera of the smartphone (A320, 2017, Samsung Group) to acquire nocturnal cough intensity and the color of patients’ sputum as an indicator for bacterial content. Finally, the air quality and smoking behavior in the bedroom is monitored (Foobot, Airboxlab, Esch sur Alzette) through modalities like temperature and humidity, particulate matters, and volatile organic compounds in the air. This integrated approach allows the initialization, setup, and testing of all devices before the handover to the patient and thus mitigates any installations and related technical issues from the patient. To assure the on-state of the Bluetooth and WiFi hotspot channel of the smartphone, a macro app (MacroDroid, developed by ArloSoft) was enabled, triggering switch-on events after a system restart or power connection events. All unnecessary apps on the phone are suppressed by an app-blocking app (Confidant, developed by Confidant Inc.).

**Figure 2:**
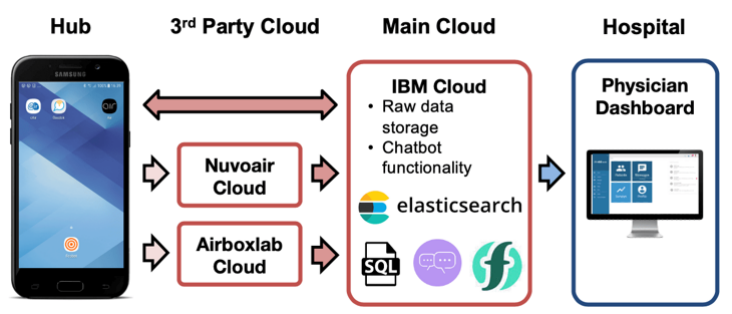
CAir data management architecture and data flow.

The CAir backend performs data collection and storage management by microservices running in the cloud (Figure 2). Patient information, device allocations, and interactions are stored in a relational database, compared to a schema-free JavaScript Object Notation object store (ElasticSearch, Lucene library) for the raw sensor data. Data are collected from the sensors in one of two ways: (1) through the third party cloud of the sensor providers and then downloaded to the CAir cloud or (2) directly from the CAir app to the CAir cloud. Patient details and daily summaries can be accessed by the study team through a web-based docdok.health physician dashboard.

Further, a CAir chatbot backend was implemented using IBM cloud functions and the IBM Watson Assistant for just-in-time interactions with the patients through a chatbot frontend provided by docdok.health. Timed triggers initiate daily conversations, such as informative sessions, physical exercises, or patient feedback through the docdok app. Further, these functions scan the databases for daily activities, degrading scores of the COPD assessment test, or nonadherence to the study protocol, notifying the study team through the docdok dashboard for health care professionals to intervene. The content and logic of the chatbot conversations are implemented using the IBM Watson Assistant, which is queried by the cloud functions to manage the conversation flow between the patient docdok app and the backend.

### Intervention

#### Control Group

Participants randomized to the control group will receive usual care (ie, physician visits every 3-6 months and therapy according to respective treatment guidelines) and a CAir Desk for 12 weeks. The CAir Desk will assess daily symptom burden, physical activity, and spirometry data. Patients will receive daily COPD assessment questionnaires through the smartphone and will be required to wear the physical activity monitor during the day and night. The threshold for sufficient physical activity and spirometry data requires measurements on at least 3 days per week. In contrast to the intervention group, participants will not receive any feedback or scores of the telemonitoring efforts, such as the daily reported COPD assessment test and daily physical activity, nor will they receive any virtual coaching.

In case of any worsening symptoms, no alert message will be generated. Patients will have to contact their general physician or pulmonologist individually and independently. No modification of any treatment will be made by the study team during the study.

#### Intervention Group

Participants randomized to the intervention group will receive a CAir Desk and a hybrid digital coaching intervention for 12 weeks.

The first week will serve to measure baseline levels of daily physical activity. Starting in the second week of the study, participants will receive individualized feedback on their daily physical activity through the CAir chatbot app. We calibrated the daily target step count to be 15% above the patient's baseline value. The telemonitoring solution will send alerts to the study team in case the patient’s daily COPD assessment test score drops by 2 or more points in 2 consecutive days. A 2-point drop is considered the minimum clinically relevant difference [16]. In those cases, a physician will contact patients by phone to discuss if any further actions have to be taken to treat or prevent a possible exacerbation (ie, taking medication or planning a physician visit).

In addition, the CAir chatbot will provide virtual coaching to the patients of the intervention group: (1) a self-paced educational component about living with COPD based on the *Living well with COPD* program [9] that aims to foster patient empowerment through engagement and awareness and (2) a dynamic motivational interviewing component to increase physical activity, one of the most critical behaviours that are of prognostic relevance. The chatbot is rule-based with predefined answer options only and does not have any anthropomorphic visual cues. We used IBM Watson as a development tool. Data gathered via the CAir Desk autonomously feeds into the chatbot interaction without time lag as a digital trigger, that is, the script of the chatbot automatically adapts if the patient has not achieved the required target step count.

During 2 time periods of the intervention, a COPD expert will grant optional additional support via chat, making the entire intervention a hybrid solution (involving the chatbot and human health care provider), which has been described in other studies [17].

**Figure 3:**
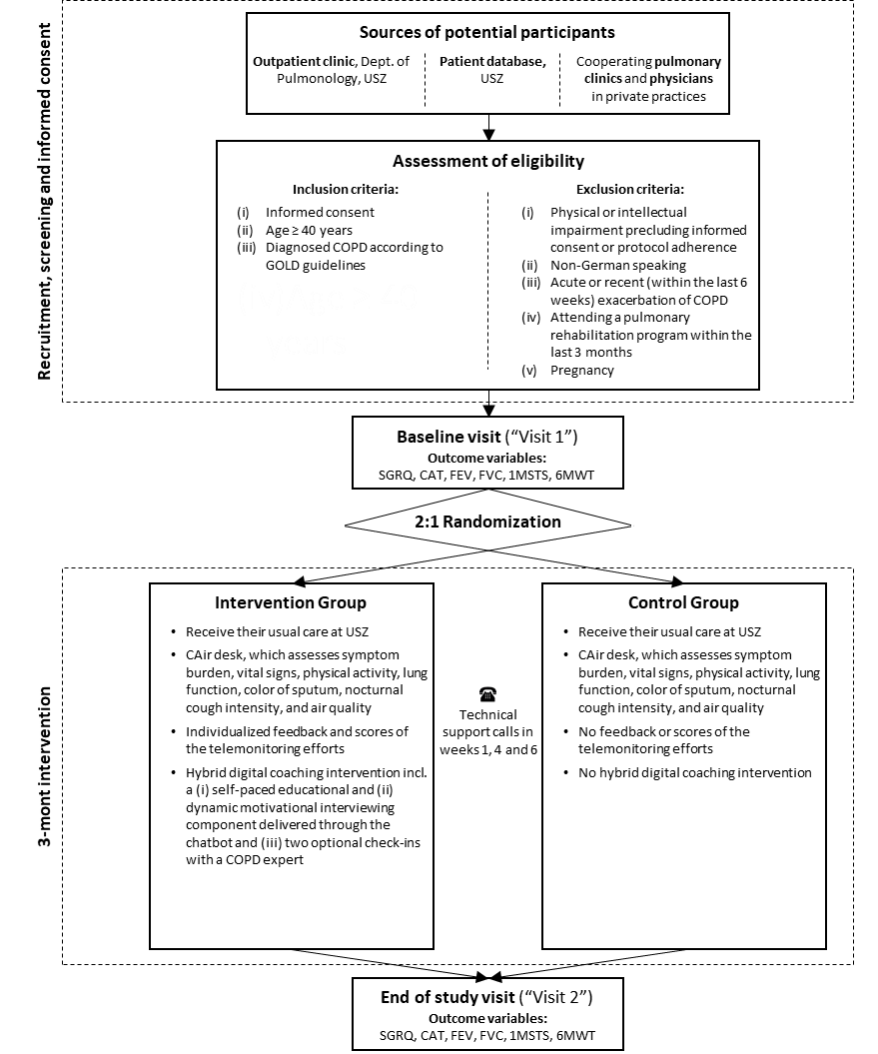
CAir Study overview.

The overall study is depicted in Figure 3.

### Outcomes

#### Primary Outcome

As a primary outcome, we will measure the delta in HRQOL, which we will assess with the SGRQ [12] from baseline to week 12 (the end of the intervention). The SGRQ is a well-established self-administered questionnaire that is valid, reliable, and responsive for the population affected by COPD. Participants will not have access to their completed questionnaires and scoring to reduce potential bias.

#### Secondary Outcomes

To evaluate the effectiveness of CAir holistically, we will include a set of secondary endpoints. Changes in the functional exercise capacity will be analyzed using the 1-minute sit-to-stand test and the 6-minute walk test, which have been demonstrated to be reliable, valid, and responsive in assessing functional exercise capacity in patients with COPD [18,19].

The 1-minute sit-to-stand test is performed using a standardized protocol [18,20]. A conventional chair without armrest and a seat height of 46 cm will be used. The patient will be instructed to stand up and sit down as often as possible at a self-chosen speed over 1 minute. The number of sit-stand repetitions will be counted by the assessor. Verbal encouragement will not be provided during the test, but the patient will be told after 45 seconds that another 15 seconds are left until the test is over. Patients will be allowed to stop at any time during the test. When standing up, the patient’s legs have to be completely straight, and when sitting down, the bottom has to have clear contact with the chair. The patients will be told to place their hands at the hips and will not be allowed to use their hands or arms to assist movement. Measurements of heart rate, peripheral capillary oxygen saturation, and modified Borg scale of perceived exertion [21] will be performed before and after the 1-minute sit-to-stand test using the same assessment tools as for the 6-minute walk test.

The 6-minute walk test will be performed according to technical standards of the American Thoracic Society/European Respiratory Society [22]. The test will be carried out on a marked circular 75-m hallway and patients will be told to walk as far as possible within the 6 minutes. The walking distance (in meters) will be registered at the end of the test. Patients will be allowed to take breaks during the test if necessary; time recording, however, will not be interrupted. Standardized instructions and phrases of encouragement will be given each minute. Oxygen supplementation will be installed if required, and the patients will carry their oxygen device during the test. Before and after the test, heart rate and oxygen saturation will be measured with a pulse oximeter (Masimo Rad-5v, Masimo Corp) connected to the index finger. Ratings of perceived exertion and dyspnea will be evaluated using a 0-10 modified Borg scale.

The COPD Assessment Test [23], a validated patient-completed questionnaire, will be provided daily on the smartphone to capture the gradual change in symptom burden.

Changes in spirometry will be measured daily with the forced expiratory volume in 1 second and forced vital capacity. Lung function testing will be performed according to the guidelines of the European Respiratory Association and the American Thoracic Society [24].

Total change in daily physical activity will be expressed as the number of steps per day. This will be assessed using a physical activity tracker. The device will be worn as a wrist band. Wearing times will be throughout day and night. During times of lower activity (eg, while having dinner, reading, or watching TV), the device may be placed on the CAir Desk, recharging its battery and synchronizing the data.

The number of recorded coughs per night will be used as a proxy for the total change in nightly cough. Compliance with the COPD medication will be captured daily and compared within and between the intervention and control group.

#### Exploratory Endpoints

Exploratory endpoints to support the analysis will also be considered. We will investigate the usability and limitations of CAir through specifically designed questionnaires (eg, usability questionnaire) and structured focus group interviews at the end of the study. In the case of an exacerbation, we will compare the data assessed by CAir before and after the event. We will also analyze air quality by continuously measuring volatile organic compounds, which are harmful organic chemicals found in products such as cleaners and in cigarette smoke. Serious adverse events related to the intervention, such as medical occurrences that require in-patient hospitalization or prolongation of existing hospitalization, will be captured as safety endpoint variables.

A detailed overview of primary, secondary, and exploratory endpoints, including their measurement frequency, is depicted in Table 1.

**Table 1:** Overview of the information collected during screening, at baseline, during the intervention and at the end of the study.

Outcome		Assessment	Cadence			
			Screening	Baseline visit	Within the intervention	End of study visit
**Primary outcome**						
	Health-related quality of life	St George Respiratory Questionnaire		✓		✓
**Secondary outcome**						
	Symptom burden	COPD^a^ assessment test		✓	Daily	✓
	Spirometry	Forced expiratory volume in one second		✓	Daily	✓
		Forced vital capacity		✓	Daily	✓
	Functional exercise capacity	1-minute sit-to-stand test		✓		✓
		6-minute walk test		✓		✓
	Compliance with COPD^a^ medication	Self-reported			Daily	
	Physical activity	Step count			Daily	
	Cough	Night-time cough count			Daily	
**Explanatory endpoints**						
	**System usability**					
		Questionnaires			Weekly	
		Focus group interviews				✓
	**Exacerbation and air quality**					
		Ex ante data		In case of event		
		Ex post data		In case of event		
		Volatile organic compounds			Continuously	

^a^COPD: chronic obstructive pulmonary disease.

### Statistical Analysis

Statistical analysis will be carried out after completing the data collection period, which will last around 18 months. We will not perform any interim analysis. We will use descriptive statistics to describe group characteristics and perform independent samples *t* tests to explore and identify potential differences at the primary endpoint (HRQOL) between the intervention and control group. We will compare the differences in the percentages of SGRQ (≥4 points) improvement between the control and intervention groups. The statistical analysis will be performed using the latest version of R (R Core Team 2019) for Windows. Missing data at the baseline and end of study visits will be handled through multiple imputation at the analysis stage. Factors prone to influence endpoints, such as gender, age, and the Global Initiative for Chronic Obstructive Lung Disease classification, will be assessed and accounted for in the analysis and interpretation of the data.

### Ethics and Dissemination

The study has received approval by the Ethics Committee of the Canton of Zurich and was registered in ClinicalTrials.gov (identifier: NCT04373070).

After the statistical analysis of this study, overall and subordinate findings will be submitted for publication in peer-reviewed journals. Dissemination of the results will be independent of negative or positive findings. The principles of the American Psychological Association guidelines for authorship eligibility will be followed.

## Results

The development of the CAir Desk and virtual coach is completed. Recruitment to the trial started in September 2020. We expect to start data collection by December 2020. This is 5 months behind schedule, which is due to the outbreak of COVID-19 that heavily impairs patients with COPD. Data collection is expected to last for approximately 18 months (first patient in, last patient out), as we follow a multiwave approach. The length of study per patient will be 12 weeks. The limited amount of available CAir Desks prevents us from executing a larger number of interventions simultaneously. We expect to complete data collection by mid-2022 and plan the dissemination of results subsequently.

## Discussion

### Study Contributions

To our knowledge, this is the first study investigating a combination of telemonitoring and hybrid virtual coaching in patients with COPD. We will investigate the effectiveness, efficacy, and usability of the proposed intervention and thus provide evidence to further develop app-based and chatbot-based disease monitoring and interventions in COPD. The study may also be extended to other respiratory diseases (eg, cystic fibrosis and COVID-19) and with an adapted technical setup to other chronic diseases requiring long-term treatment (eg, diabetes mellitus type 2 and chronic kidney disease).

### Strengths and Limitations

Randomized controlled trials are considered the “gold standard for evaluating efficacy in clinical research and constitute evidence for medical treatment” [25]. By adopting such a trial and ensuring internal as well as external validity, we maximize the robustness of our study. To make sure that the CAir Desk will function well with the study population, we extensively pretested the device in patients with COPD. The technology we used as part of the CAir Desk is well established and not likely to malfunction in daily use. A possible limitation is that the intervention is rather technology-heavy and the study population includes persons aged 45 years and older. Even though we pretested the system usability extensively in patients with COPD from the same population, some participants might feel overwhelmed and therefore discontinue the study. In addition, patients may feel overloaded by the daily measurements and questionnaires to be answered, which could also lead to dropouts. Another possible limitation is the limited size of the sample, given the heterogeneity in the population affected by COPD.

## Appendix

Multimedia Appendix 1 Grant letter from Innosuisse.

## Abbreviations

COPD: chronic obstructive pulmonary disease
HRQOL: health-related quality-of-life
SGRQ: St. George Respiratory Questionnaire
