# Supplementary material for: A Telemonitoring and Hybrid Virtual Coaching Solution “CAir” for Patients with Chronic Obstructive Pulmonary Disease: Protocol for a Randomized Controlled Trial
Source: JMIR Res Protoc. 2020 Oct 22;9(10):e20412. doi: 10.2196/20412 (PMC7644383; doi:10.2196/20412)
Supplement: Multimedia Appendix 1 [file resprot_v9i10e20412_app1.pdf]

Schweizerische Eidgenossenschaft
Confédération suisse
Confederazione Svizzera
Confederaziun svizra

Innosuisse – Schweizerische Agentur für Innovationsförderung

CH-3003 Bern, Innosuisse

A-Post
USZ
Mr. Christian Clarenbach
Rämistrasse 100
8091 Zürich

Our ref.: JOD
Bern, 24.10.2018

Innosuisse no. 29844.1 IP-ICT / project agreement

Dear Mr. Clarenbach,

We are looking forward to helping you implement your innovation project.
As you can see from the attached contract, the amount of the financial contribution to the research partner(s) was in accordance with your application and the contribution was set at CHF 49'709.

By calculating the funding budget based on the salary tariffs reported by the research partner, the financial contribution of the implementation partner increases in percentage to 15%. Innosuisse accepts a reduction of the cash contribution if the research partner(s) agree and the amount of the financial contribution of at least 10% of the funding budget remains guaranteed. In this case, please apply for a corresponding reduction by sending an e-mail to ict@innosuisse.ch. All project partners involved must confirm the request

Enclosed you will find the agreement, which stipulates the conditions for payment of the Innosuisse project funding and details of the partnership. Please check the provisions through carefully and send a copy signed by all project partners back to us at the following address:

Innosuisse – Swiss Innovation Promotion Agency
Innovation projects
Einsteinstrasse 2
CH-3003 Bern

Please, inform us about the definitive project starting date by e-mail (you can find the e-mail address below).

Team ICT
Tel. +41-58 467 17 55
E-Mail ict@innosuisse.ch
Innosuisse
Einsteinstrasse 2, 3003 Bern
www.innosuisse.ch

We will initiate the payment of the first tranche as soon as we receive the signed agreement. We wish you and your partners every success with the project. Please do not hesitate to contact us if you have any questions.

Kind regards

Dannie Jost
Scientific Officer

Enclosures:
5 copies of the agreement
